# Probucol Increases Striatal Glutathione Peroxidase Activity and Protects against 3-Nitropropionic Acid-Induced Pro-Oxidative Damage in Rats

**DOI:** 10.1371/journal.pone.0067658

**Published:** 2013-06-14

**Authors:** Dirleise Colle, Danúbia Bonfanti Santos, Eduardo Luiz Gasnhar Moreira, Juliana Montagna Hartwig, Alessandra Antunes dos Santos, Luciana Teixeira Zimmermann, Mariana Appel Hort, Marcelo Farina

**Affiliations:** Departamento de Bioquímica, Centro de Ciências Biológicas, Universidade Federal de Santa Catarina, Florianópolis, Santa Catarina, Brazil; Federal University of Rio de Janeiro, Brazil

## Abstract

Huntington’s disease (HD) is an autosomal dominantly inherited neurodegenerative disease characterized by symptoms attributable to the death of striatal and cortical neurons. The molecular mechanisms mediating neuronal death in HD involve oxidative stress and mitochondrial dysfunction. Administration of 3-nitropropionic acid (3-NP), an irreversible inhibitor of the mitochondrial enzyme succinate dehydrogenase, in rodents has been proposed as a useful experimental model of HD. This study evaluated the effects of probucol, a lipid-lowering agent with anti-inflammatory and antioxidant properties, on the biochemical parameters related to oxidative stress, as well as on the behavioral parameters related to motor function in an *in vivo* HD model based on 3-NP intoxication in rats. Animals were treated with 3.5 mg/kg of probucol in drinking water daily for 2 months and, subsequently, received 3-NP (25 mg/kg i.p.) once a day for 6 days. At the end of the treatments, 3-NP-treated animals showed a significant decrease in body weight, which corresponded with impairment on motor ability, inhibition of mitochondrial complex II activity and oxidative stress in the striatum. Probucol, which did not rescue complex II inhibition, protected against behavioral and striatal biochemical changes induced by 3-NP, attenuating 3-NP-induced motor impairments and striatal oxidative stress. Importantly, probucol was able to increase activity of glutathione peroxidase (GPx), an enzyme important in mediating the detoxification of peroxides in the central nervous system. The major finding of this study was that probucol protected against 3-NP-induced behavioral and striatal biochemical changes without affecting 3-NP-induced mitochondrial complex II inhibition, indicating that long-term probucol treatment resulted in an increased resistance against neurotoxic events (i.e., increased oxidative damage) secondary to mitochondrial dysfunction. These data appeared to be of great relevance when extrapolated to human neurodegenerative processes involving mitochondrial dysfunction and indicates that GPx is an important molecular target involved in the beneficial effects of probucol.

## Introduction

Huntington’s disease (HD) is a neurodegenerative disorder primarily caused by a mutation in the gene encoding Huntingtin, which results in the production of a mutated protein (mHtt) [1]. mHtt modulates molecular events that are responsible for the progressive neurodegeneration of the caudate nucleus and putamen in the basal ganglia [2] and in cortical regions [3], which manifests with cognitive disturbance, behavioral disorders, and movement incoordination [1].

A large body of evidence from both experimental and clinical studies supports a pivotal role for oxidative stress and attendant mitochondrial dysfunction in mediating the neuronal degeneration observed in HD [4]. Increased levels of oxidative damage products, including protein nitration, lipid peroxidation, DNA oxidation, and exacerbated lipofuscin accumulation, occur in HD [5,6]. Importantly, oxidative stress and mitochondrial dysfunction are connected phenomena that feed off each other in HD, leading to a vicious cycle of energy deficits that culminates in neurodegeneration [4].

The administration of 3-nitropropionic acid (3-NP) in rodents and non-human primates has been proposed as a useful experimental model of HD; both biochemical and behavioral characteristics observed in HD patients are reproduced in this model [7]. The primary mechanism of 3-NP-induced neurotoxicity involves the irreversible inhibition of succinate dehydrogenase (SDH), a key enzyme located at the inner mitochondrial membrane and responsible for the conversion of succinate into fumarate [7,8]. SDH inhibition interferes with the mitochondrial electron transport cascade and oxidative phosphorylation, which results in a cellular energy deficit [9]. However, there is evidence that impaired electron transference via the mitochondrial electron chain results in an increased generation of reactive oxygen (ROS) and nitrogen (RNS) species [10], which are critically involved in 3-NP-induced oxidative stress and neuronal death.

Given the importance of oxidative stress in HD, several experimental antioxidant and bioenergetic strategies have been employed in HD mice models, some with promising parallels in human clinical trials, and support antioxidant approaches for the treatment of HD [11,12]. Nonetheless, clinical studies have failed to show some benefits of antioxidants on the progression of symptoms in individuals with HD.

Probucol, a phenolic lipid-lowering agent with antioxidant and anti-inflammatory properties [13], has been clinically used during the past few decades for the treatment and prevention of cardiovascular diseases [13,14].

However, two adverse effects (decreased high-density lipoprotein cholesterol levels and changes in the cardiac electrophysiological homeostasis) observed in patients who have taken it for long-term periods resulted in its removal from use in several countries [15–17]. However, probucol is still used as a lipid-lowering agent in Japan, particularly for the treatment of familial hypercholesterolemia [14]. Currently, there are controversial opinions concerning the adverse effects of probucol [14,15,17], which indicate that additional studies on the toxicological and beneficial effects of probucol are warranted.

Interestingly, previous experimental studies have reported that probucol has protective effects in experimental models of neurotoxicity/neuropathology [18,19]. In addition, this compound was able to modulate oxidative stress and excitotoxicity in an *in vitro* HD model by decreasing 3-NP-induced ROS production and lipid peroxidation in striatal slices [20] and isolated mitochondria [21]. Of particular importance, an *in vitro* study from our group showed that probucol increased glutathione peroxidase (GPx) activity in primary cultures of cerebellar neurons [18], which provided a protective effect against the toxicity elicited by methylmercury (MeHg), an environmental pollutant whose mechanisms of toxicity are related, at least partially, to the increased production [22] and decreased detoxification [23] of peroxides. Nevertheless, data on the relationship between probucol and GPx activity under *in vivo* conditions are scarce.

As previously mentioned, oxidative stress represents a crucial event in HD experimental models [24,25], as well as in the pathogenesis of HD [6,26], and glutathione peroxidase has been demonstrated as an important enzyme in this scenario [25,26]. On the basis of this evidence and on the previously reported modulating effect of probucol toward GPx under *in vitro* conditions [18], we hypothesized that this compound could present beneficial effects in an *in vivo* HD model based on 3-NP intoxication. Our research was motivated by previous data that support the rationale for therapeutic strategies that either potentiate antioxidant defenses or avoid oxidative stress generation to delay HD progression [26].

## Methods

### Chemicals

Probucol, 3-nitropropionic acid, β-nicotinamide adenine dinucleotide phosphate sodium salt reduced form, glutathione reductase from baker’s yeast, reduced glutathione and dimethyl sulfoxide were obtained from Sigma (St. Louis, MO, USA). Rabbit polyclonal IgG anti-inducible nitric oxide synthase (iNOS: sc-8310), mouse monoclonal IgG anti-glial fibrillary acidic protein (GFAP: sc-166481), rabbit polyclonal IgG anti-glutathione peroxidase protein (GPx-1: sc 30147), rabbit polyclonal IgG anti-Mn-superoxide dismutase protein (Mn–SOD/SOD 2: sc 30080), mouse monoclonal anti-β-actin primary antibody (β-actin: sc-47778) and protein A/G horseradish peroxidase-conjugated secondary antibody were purchased from Santa Cruz (Santa Cruz, CA). Rabbit polyclonal IgG anti-caspase 3 (9662S-detects endogenous levels of full-length caspase-3 and the large fragment of caspase-3 resulting from cleavage) and rabbit polyclonal IgG anti-CuZn-superoxide dismutase protein (CuZn–SOD/SOD 1: 27705) were obtained from Cell Signaling Technology (USA). All other chemicals were of the highest grade available commercially.

### Animals

Adult Wistar male rats (3 months old) were obtained from the animal facility of the Universidade Federal de Santa Catarina (UFSC, Florianópolis, Brazil). Animals were maintained at 22°C, on a 12 h light: 12 h dark cycle, with free access to food and water. All of the experiments were performed in accordance with the Guiding Principles in the Use of Animals in Toxicology, adopted by the Society of Toxicology (1989), and the protocols used were approved by our ethics committee for animal use at the Universidade Federal de Santa Catarina (CEUA/UFSC PP00424; 23080.008706/2010-52).

### Drug treatment protocol

Forty animals were randomly divided into four groups consisting of 10 animals each, as follows: (1) Control, (2) Probucol, (3) 3-NP and (4) Probucol + 3-NP. Animals from groups 2 and 4 received probucol (40 mg/L) diluted in a 1% DMSO solution *ad libitum*, as the sole source of liquid for 2 months. Groups 1 and 3 were treated with vehicle (1% DMSO, *ad libitum*) for 2 months. For probucol-treated rats (groups 2 and 4), a daily probucol dose of 3.5 mg/kg of body weight was calculated based on their daily liquid ingestion (30.05 ± 0.031 mL/animal). The liquid ingestion of these animals (groups 1-4) was not different from a parallel group of animals that received only tap water (data not shown). The dose of 3.5 mg/kg/day was based on that normally given to humans (250 mg/day), which represents a dose of 3.57 mg/kg/day when considering a body mass of 70 kg. It is also important to mention that the 2-month treatment was not sufficient to significantly decrease plasma cholesterol levels in the animals (data not shown), which were normocholesterolemic at the beginning of the treatments.

Two months after the beginning of probucol treatment, the animals received 25 mg/kg of 3-NP intraperitoneally (i.p.) or vehicle (NaCl 0.9%), once a day for six consecutive days [27,28] with continuous treatment in the drinking water (probucol or vehicle). Weight gain was monitored every 2 weeks.

### Behavioral analysis

Twenty-four hours after the last 3-NP administration, animal were submitted to behavioral analysis. The open field task was performed to evaluate spontaneous locomotor activity of the rats. The animals were evaluated for 5 min in an open field arena [29]. The apparatus, made of wood and covered with impermeable formica, had a 100 cm x 100 cm white floor (divided by black lines into 25 cm x 20 cm squares) and 40-cm high white walls. Each rat was placed in the center of the open field, and the number of squares crossed and the number of rearings was recorded [30].

The integrity of the motor system was evaluated using the rotarod test. Briefly, the rotarod apparatus consisted of a rod 30 cm long and 3 cm in diameter that was subdivided into four compartments by discs 24 cm in diameter. The rod rotated at a constant speed of 14 rpm. The animals were given a prior training session before the initialization of 3-NP administration to acclimate the animals to the apparatus. The latency for first fall from the rod and the number of falls were recorded. The cut-off time was 240 s [31].

### Tissue preparation for biochemical analysis

Twenty-four hours after the behavioral analyses, the animals were euthanized by decapitation, the brain was removed and the striatum was dissected. The striatum (from the right hemisphere) of twenty-four animals (6 per group) were randomly homogenized (1: 10 w/v) in HEPES buffer (20 mM, pH 7.0). The tissue homogenates were centrifuged at 16,000 x *g* and 4°C for 20 min, and the supernatants obtained were used for the determination of enzymatic activities, and the quantification of the levels of reduced glutathione (GSH) and thiobarbituric acid reactive substances (TBARS).

### Antioxidant enzymes

Striatal glutathione reductase (GR) activity was determined based on the protocol developed by Carlberg and Mannervik [32]. Briefly, GR reduces GSSG to GSH at the expense of NADPH; the disappearance of NADPH can be detected at 340 nm. Striatal glutathione peroxidase (GPx) activity was determined based on the protocol developed by Wendel [33] by indirectly measuring the consumption of NADPH at 340 nm. GPx uses GSH to reduce tert-butyl hydroperoxide, thereby producing GSSG, which is readily reduced to GSH by GR using NADPH as a reducing equivalent donor.

Superoxide dismutase (SOD) activity was determined in striatal homogenates according to Misra and Fridowich in 480 nm [34]. The addition of tissue samples (5, 10, and 20 µL) containing SOD inhibits the auto-oxidation of epinephrine. The rate of inhibition was monitored for 180 seconds. The amount of enzyme required to produce 50% inhibition was defined as one unit of enzyme activity. Catalase activity was measured according to the method of Aebi [35]. The reaction was initiated by the addition of freshly prepared 30 mM H_2_O_2_. The rate of H_2_O_2_ decomposition was measured spectrophotometrically at 240 nm.

### Reduced glutathione levels

Reduced glutathione (GSH) levels were determined using a fluorimetric assay as previously described [36]. GSH was measured in striatal homogenates after precipitation with 1 volume of 0.6 M perchloric acid and centrifuged at 14,000 rpm at 4^°^C for 10 min. A volume of 50 µL of supernatant was incubated with 100 µL of ortho-phthaldehyde (0.1% w/v in methanol) and 1.85 ml of 100 mM Na_2_HPO_4_ for 15 min at room temperature. The fluorescence intensity was read in a microplate reader (with excitation and emission wavelengths of 350 nm and 420 nm, respectively). The GSH content was calculated using standard curves that were run concurrently and expressed as nmol GSH·mg protein^-1^.

### Determination of thiobarbituric acid reactive substance levels

Thiobarbituric acid reactive substances (TBARS) were determined in the striatal homogenates using the method described by Ohkawa and colleagues [37], in which malondialdehyde (MDA), an end-product of lipid peroxidation, reacts with thiobarbituric acid to form a colored complex. The samples were incubated at 100°C for 60 minutes in acid medium containing 0.45% sodium dodecyl sulfate and 0.67% thiobarbituric acid. After centrifugation, the reaction product was determined at 532 nm using MDA as a standard.

### Respiratory chain complex II activity

The striatum (from the left hemisphere) was homogenized (1: 10 w/v) in 4.4 mM potassium phosphate buffer, pH 7.4, containing 0.3 M sucrose, 5 mM MOPS, 1 mM EGTA and 0.1% bovine serum albumin. The homogenates were centrifuged at 3,000 × *g* for 10 min at 4°C. The pellet was discarded and the supernatants were centrifuged at 17,000 × *g* for 10 min at 4°C. The obtained pellet was dissolved in the same buffer and kept at -70°C until enzymatic activity determination [38].

The activity of succinate-2,6-dichloroindophenol (DCIP)-oxidoreductase (complex II) was determined according to the method of Fischer and colleagues [39]. Complex II activity was measured by following the decrease in absorbance due to the reduction of 2,6-DCIP at 600 nm and calculated as nmol.min^−1^.mg protein^−1^.

### Western blotting analyses

The striatal tissues of sixteen animals (4 per group) were homogenized (1: 10 w/v) in ice-cold lysis buffer (50 mM Tris-HCl, pH 7.5, 1% Triton X-100, 100 mM NaCl, 5 mM EDTA, pH 8.0, 40 mM β-glycerolphosphate, 50 mM NaF, 200 µM orthovanadate, 5% glycerol and protease inhibitors). The homogenates were centrifuged at 13,000 x *g*, at 4°C for 45 min. Prior to western blotting analysis, equivalent amounts of proteins were mixed in buffer (200 mM Tris, 10% glycerol, 2% SDS, 2.75 mM β-mercaptoethanol and 0.04% bromophenol blue), boiled for 5 minutes and stored at −20°C until further western blot analyses.

Fifty micrograms of protein extract was subjected to SDS polyacrylamide gel electrophoresis (PAGE) using 7.5% and 12% gels, and the proteins were transferred onto nitrocellulose membranes using a tank transfer system at 100 V and 400 mA for 90 min. The membranes were blocked (1 h) with 5% skim milk in TBS (10 mM Tris, 150 mM NaCl, pH 7.5). The blots were incubated overnight at 4^°^C with anti-iNOS antibody (1:1000; 130 kDa), anti-GFAP (1:3000; 50 kDa), anti-caspase 3 total/cleaved caspase 3 (1:1000; 35 and 17 kDa), SOD-1 (1:1000; 20 kDa), SOD-2 (1:500; 25 kDa), GPx-1 (1:250; 22 kDa) or anti-β-actin (1:2000; 43 kDa) in TBS-Tween-BSA buffer (20 mM Tris base, 140 mM NaCl, 0.05% Tween-20, 2% BSA). After several washes, the blots were incubated for 60 min at room temperature with protein A/G-horseradish peroxidase conjugate in TBS-Tween buffer. Next, the membranes were washed and developed with Immun-Star HRP Chemiluminescent reagents (Luminol Reagent sc-2048, Santa Cruz, CA, USA), and chemiluminescence was detected using the ECL System. β-actin was used as a loading control. The band intensities were quantified using the Scion Image software (Scion Corporation, Frederick, MD, USA). The densitometric values from the iNOS, GFAP, caspase 3, GPx-1 and SOD 1 and 2 bands were normalized with respect to the β-actin band.

### Protein determination

The protein content measurements were assessed according to the Lowry method [40].

### Statistical analysis

Data were analyzed using the STATISTICA software system, version 8.0 (StatSoft, Inc., 2008). Differences among the groups and/or 3-NP vs. probucol interactions were analyzed by two-way ANOVA followed by the Tukey post hoc test. The results are expressed as the mean ± SEM. Differences were considered significant when p< 0.05. All of the graphics were generated using the GraphPad Prism (GraphPad Software, San Diego, CA, USA).

## Results

### Probucol protects against 3-NP-induced decrease in body weight but does not modify complex II inhibition

Probucol treatment did not affect any physiological parameters such as body weight and food or liquid consumption compared to non-treated animals prior to 3-NP administration (data not shown).

**Figure 1:**
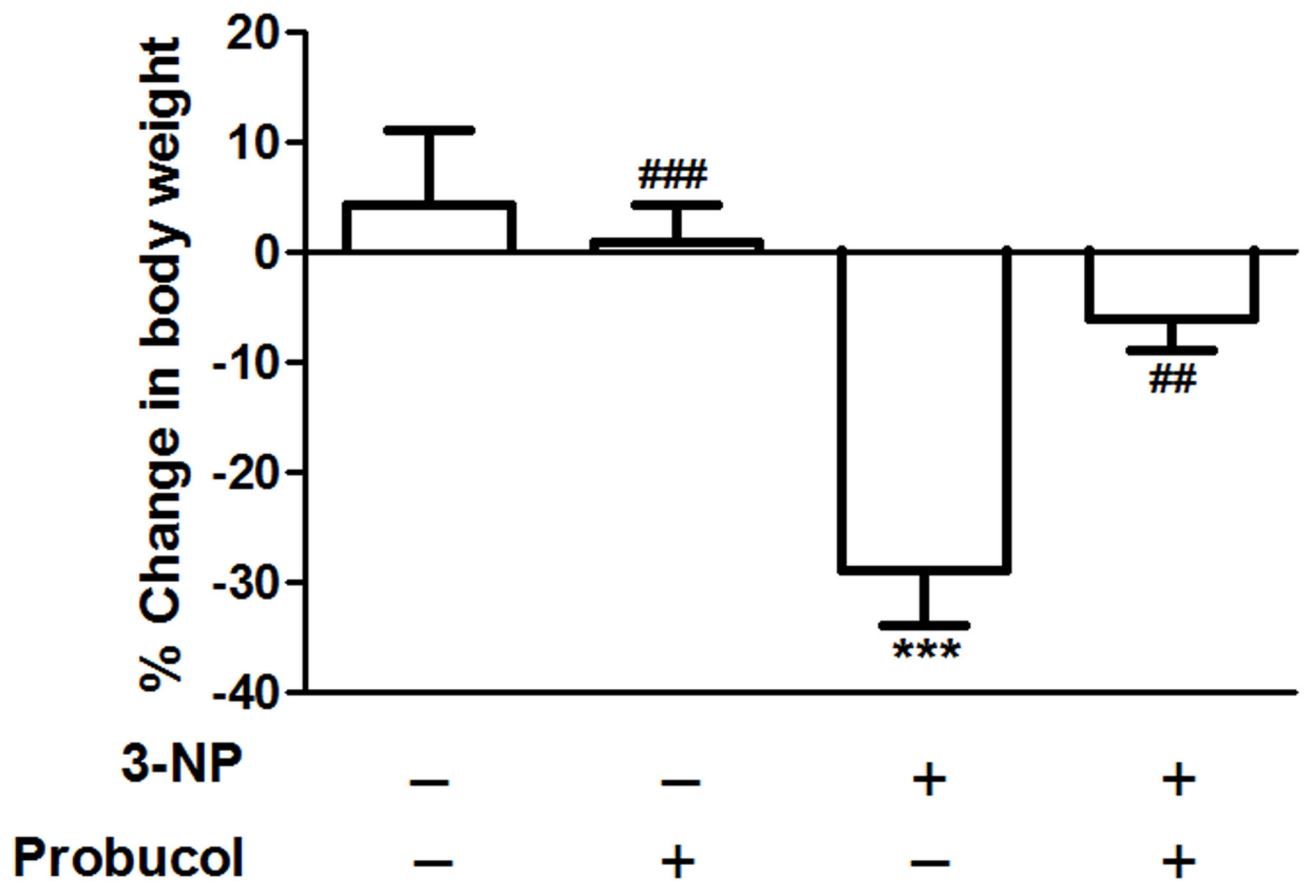
Probucol prevents 3-NP-induced decreases in body weight. The animals were pretreated with probucol (3.5 mg/kg/day) or vehicle (1% of DMSO) in drinking water daily for 2 months and administered intraperitoneally with 3-NP (25 mg/kg) or vehicle, once a day for 6 consecutive days. The body weight values are expressed as the percentage of change in body weight after 3-NP or vehicle administration and presented as the mean ± S.E.M. (n = 10 rats/group). ***p < 0.001 compared with the control group and ## p < 0.01 and ### p < 0.001 compared with the 3-NP group using two-way analysis of variance (ANOVA) followed by Tukey’s multiple comparison test.

Administration of 3-NP induced a significant decrease in body weight (p<0.001); however, this decrease was not observed in the animals pretreated with probucol (Figure 1). Moreover, significant 3-NP versus probucol interaction [F_(1,36)_ = 7.82; p < 0.01] was observed, indicating that probucol treatment significantly mitigated the decrease in body weight induced by 3-NP administration.

**Figure 2:**
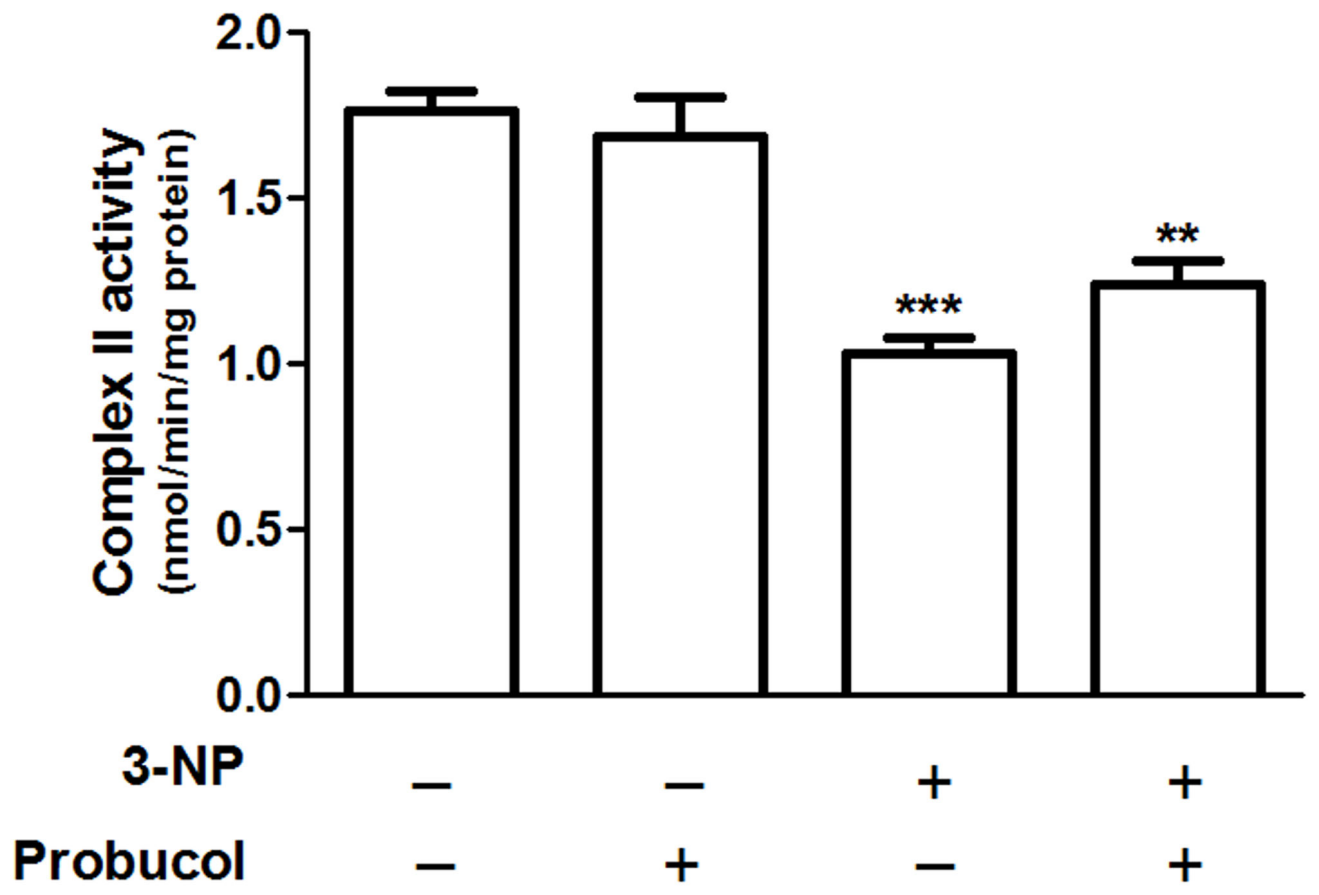
3-NP treatment inhibits complex II activity. Treatments were conducted as previously mentioned (see Methods Section). Complex II activity in striatum is expressed as nmol.min^−1^.mg protein^−1^ and presented as the mean ± S.E.M. (n = 6 rats/group). ** p < 0.01 and *** p < 0.001 compared with the control group using two-way analysis of variance (ANOVA) followed by Tukey’s multiple comparison test.

As expected, striatal mitochondrial complex II activity was inhibited in 3-NP-treated animals. A significant main effect of 3-NP factor [*F*_(1,20)_ = 39.96; p < 0.001] on complex II activity was also observed. However, this event was not modified by probucol treatment according to the non-significant probucol by 3-NP interaction [*F*_(1,20)_ = 4.4; p = 0.116] (Figure 2).

### Probucol attenuates motor impairment induced by 3-NP

**Figure 3:**
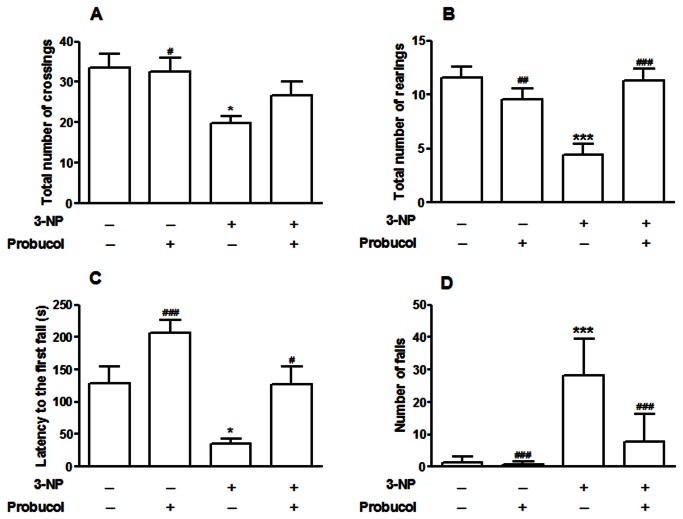
Probucol attenuates motor impairment induced by 3-NP in rats. Treatments were conducted as previously mentioned (see Methods Section). Locomotor (A) and exploratory (B) activities in the open field test as well as the latency for the first fall (C) and the number of falls in the rotarod (D) were evaluated 24 h after the last 3-NP administration. These results are expressed as the total number of crossings (A), total number of rearings (B), the latency for the first fall(s) (C) and the total number of falls. The data are presented as the mean ± S.E.M. (n =10 rats/group). *p < 0.05 and *** p < 0.001 compared with the control group and # p < 0.05, # # p < 0.01 and # # # p < 0.001 compared with the 3-NP group using two-way analysis of variance (ANOVA) followed by Tukey’s multiple comparison test.

To evaluate the effects of 3-NP administration on motor performance, open field and rotarod tasks were performed. 3-NP treatment was associated with significant alterations in the behavioral tests, which were characterized by a decrease in the number of crossings and rearings in the open field test (*p* < 0.05 and *p* < 0.001, respectively; Figure 3A and B). Two-way ANOVA indicated a significant main effect of 3-NP factor in the number of crossings [*F*_(1,36)_ = 10.23; *p* < 0.01] and rearings [*F*_(1,36)_ = 6.3; *p* < 0.01], respectively. Two-way ANOVA also indicated a significant 3-NP versus probucol interaction [*F*_(1,36)_ = 17.51; *p* < 0.001] and in the number of rearings (Figure 3B). Probucol completely protected against the 3-NP-induced decrease in the number of rearings in the open field test.

Furthermore, 3-NP treatment also induced a decrease in the latency to the first fall, and an increase in the number of falls in the rotarod task (*p* < 0.05 and *p* < 0.001, respectively; Figure 3C and D). Two-way ANOVA indicated a significant main effect of 3-NP toward the latency to the first fall [*F*_(1,33)_ = 14.77; *p* < 0.001] and the number of falls [*F*_(1,33)_ = 54.53; *p* < 0.001]. Probucol was able to improve the performance of the 3-NP-exposed rats in the rotarod task. A significant 3-NP versus probucol interaction [*F*_(1,33)_ = 8.86; *p* < 0.001] in the number of falls was observed.

### Probucol attenuates striatal oxidative stress induced by 3-NP

**Figure 4:**
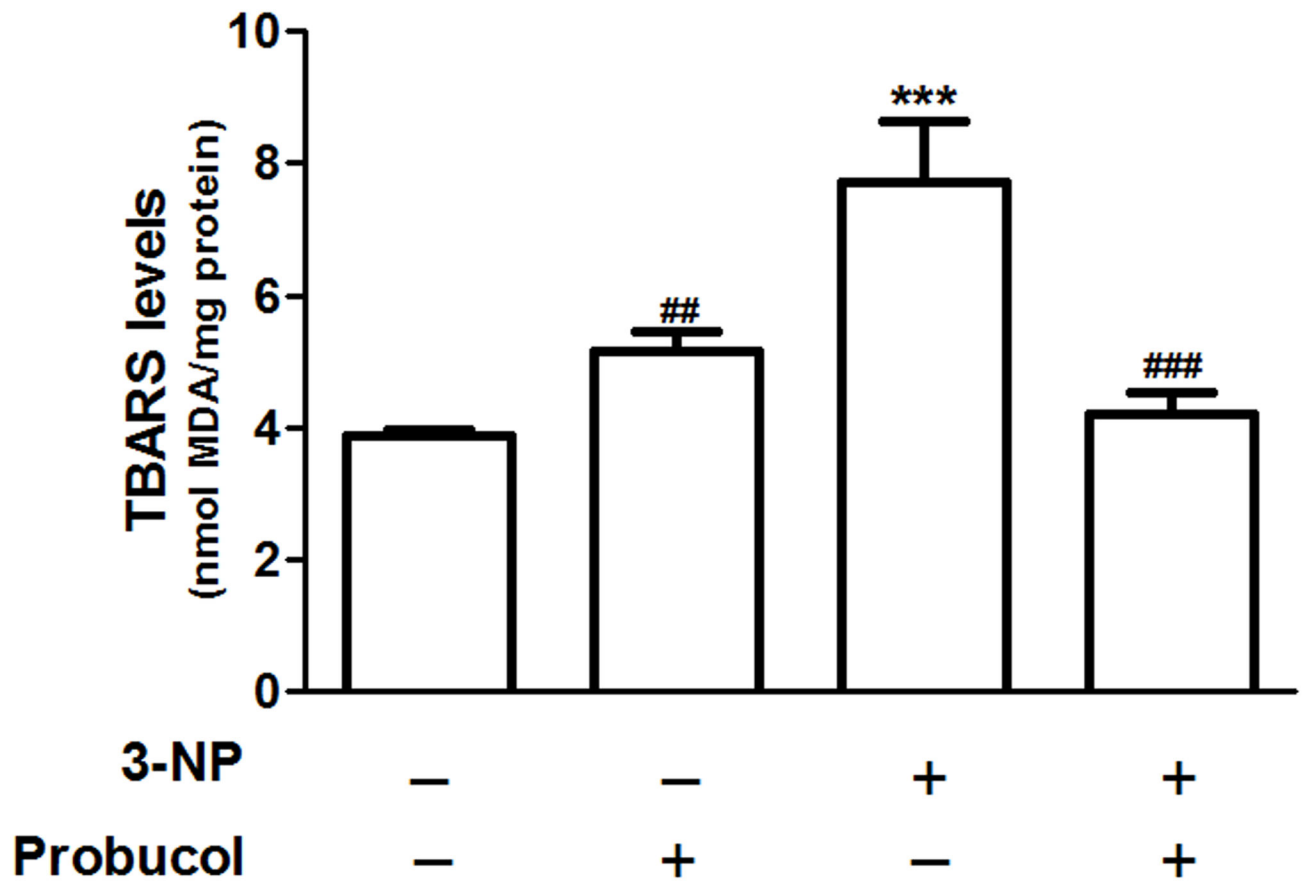
Probucol reduces 3-NP-induced lipid peroxidation in rats. Treatments were conducted as previously mentioned (see Methods Section). Striatal thiobarbituric acid reactive substance (TBARS) levels are expressed as nmol of MDA/mg protein. The data are presented as the mean ± S.E.M. (n = 6 rats/group). *** p< 0.001 compared with the control group and # # p < 0.01 and # # # p < 0.001 compared with the 3-NP group using two-way analysis of variance (ANOVA) followed by Tukey’s multiple comparison test.

As shown in Figure 4, 3-NP administration caused a significant increase in TBARS production in the striatum (p < 0.001, Figure 4). Probucol was able to blunt the effect of 3-NP-induced lipid peroxidation. Two-way ANOVA indicated a significant main effect for 3-NP by the interaction between probucol and the TBARS levels in the striatum [F_(1,20)_ = 22.58; p < 0.001].

**Figure 5:**
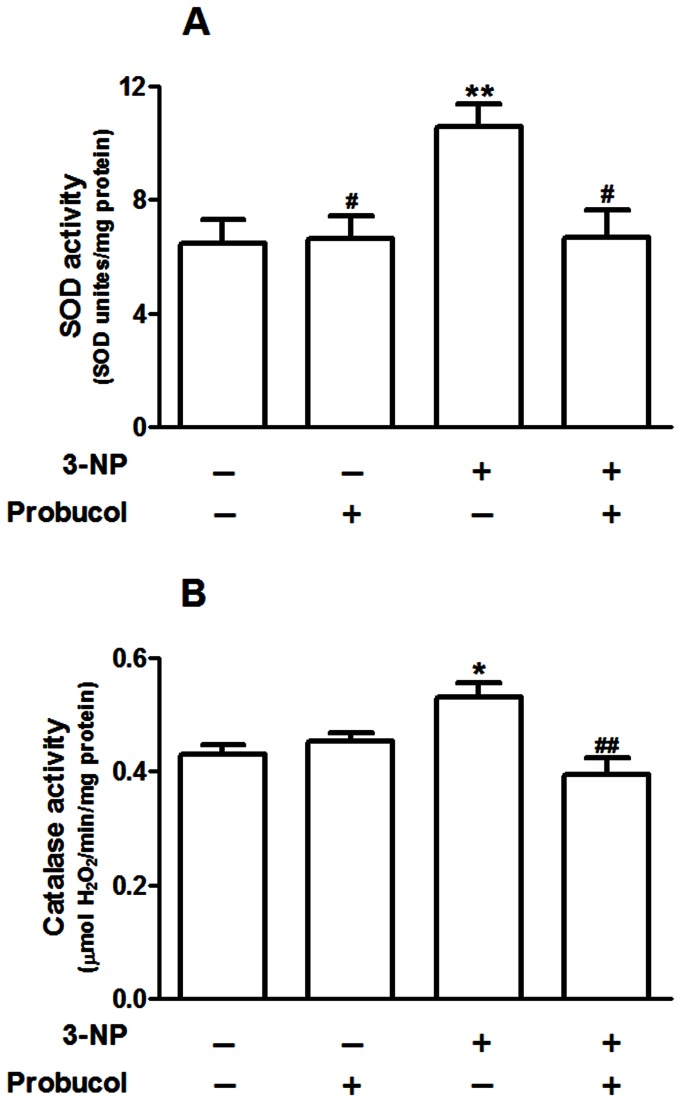
Probucol attenuates the increase in superoxide dismutase (SOD) and catalase activities in the rat striatum. Treatments were conducted as previously mentioned (see Methods Section). SOD activity (A) is expressed as SOD units/mg of protein. Catalase activity (B) is expressed as µmol of H_2_O_2_/min/mg protein. The data are presented as the mean ± S.E.M. (n = 6 rats/group). * p < 0.05 and ** p < 0.01 compared with the control group, and # p < 0.05 and # # p < 0.01 compared with the 3-NP group using two-way analysis of variance (ANOVA) followed by Tukey’s multiple comparison test.

In addition, the activities of the antioxidant enzymes, SOD and catalase, were significantly increased by 3-NP in the striatum compared to the control group (p < 0.01 and p < 0.05; Figure 5A and B, respectively). Probucol treatment significantly attenuated the 3-NP-induced increase in SOD and catalase activities. Interactions were observed between 3-NP versus probucol by two-way ANOVA on SOD [F_(1,20)_ = 6.0; p < 0.05] and catalase [F_(1,20)_ = 12.98; p < 0.01] activities. Western blotting analyses for SOD 1 and SOD 2 were performed to better understand the mechanisms mediating the 3-NP-induced oxidative stress and the protective effects afforded by probucol. The treatments did not significantly change the levels of SOD 1 and SOD 2 (Figure S1A, B and C).

**Figure 6:**
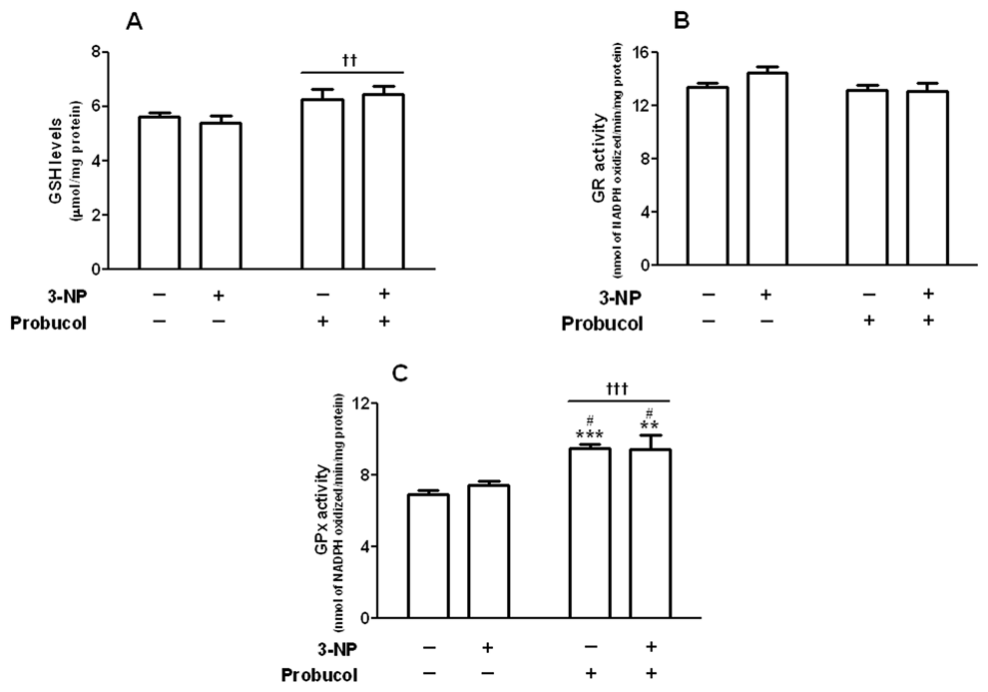
Effects of 3-NP and/or probucol on striatal GSH levels, glutathione peroxidase and glutathione reductase activities. Treatments were conducted as previously mentioned (see Methods Section). The GSH levels (A) are expressed as µmol GSH·mg protein^-1^. GR activity (B) and GPx activity (C) are expressed as the nmol of NADPH oxidized/min/mg protein. The data are presented as the mean ± S.E.M. (n= 6 rats/group). †† p < 0.01 and † † † p < 0.001 main effect of probucol, ** p < 0.01 and *** p < 0.001 compared with the control group and # p < 0.05 compared with the 3-NP group using two-way analysis of variance (ANOVA) followed by Tukey’s multiple comparison test.

Striatal GSH content was not significantly modified by 3-NP treatment (Figure 6A). However, two-way ANOVA indicated a significant main effect of probucol toward GSH levels [*F*_(1,20)_ = 9.75; *p* < 0.01]. Moreover, striatal GR activity was not significantly different among groups (Figure 6B).

Notably, probucol treatment caused a significant increase in striatal GPx activity independent of 3-NP administration (Figure 6C). Two-way ANOVA indicated a significant main effect of probucol [*F*_(1,20)_ = 29.33; *p* < 0.001) toward GPx activity. Post-hoc analyses indicated that striatal GPx activity was significantly higher in probucol and 3-NP plus probucol-exposed rats compared with the control (p < 0.001 and p < 0.01, respectively) or 3-NP-exposed animals (*p* < 0.05) (Figure 6C). However, the levels of the enzyme GPx-1, which is present in several cellular compartments and represents the main GPx isoform detoxifying hydrogen peroxide within the intracellular milieu, were not changed by probucol treatment (Figure S1D and E), which was consistent with our previous findings [18].

### Probucol treatment attenuates striatal increases in iNOS levels induced by 3-NP

**Figure 7:**
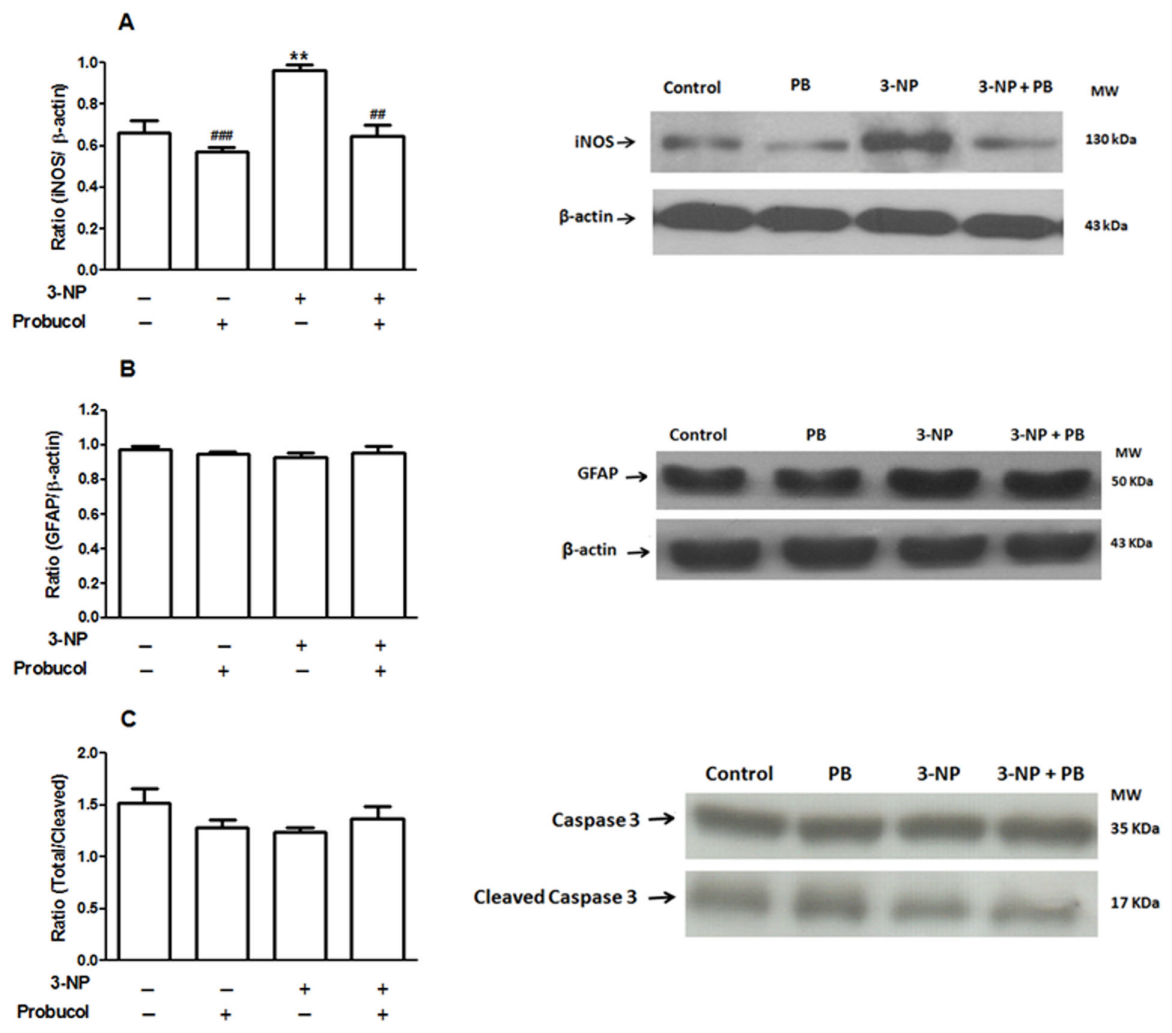
**Effects of 3-NP and/or probucol on iNOS, GFAP** and **caspase 3/cleaved caspase 3 expression.**

3-NP treatment induced a significant increase in iNOS immunoreactivity in the striatum, which was completely attenuated by probucol treatment (p < 0.01) (Figure 7A). Moreover, a significant interaction was observed between 3-NP versus probucol using two-way ANOVA on iNOS levels [F_(1,12)_ = 6.53; p < 0.05]. However, 3-NP administration was unable to significantly change the levels of GFAP (a marker of gliosis) and caspase 3 (a marker of apoptosis) (Figure 7B and C).

Treatments were conducted as previously mentioned (see Methods Section). iNOS (A), GFAP (B) and caspase 3/cleaved caspase 3 expressions were determined using Western blot analyses and expressed as the optical density compared to β-actin. The data are presented as the mean ± S.E.M. (n= 4 rats/group). ** p < 0.01 compared with the control group and # # p < 0.01 and # # # p < 0.001 compared with the 3-NP group using two-way analysis of variance (ANOVA) followed by Tukey’s multiple comparison test.

## Discussion

In the present study, we examined the effects of probucol, a lipid-lowering drug with anti-inflammatory and antioxidant properties, on the neurotoxicity and oxidative stress induced by 3-NP administration, which is a well-known experimental model of HD. Our results showed that sub-hypocholesterolemic probucol treatment protected against behavioral and striatal biochemical changes induced by i.p. administrations of 3-NP in rats, significantly attenuating 3-NP-induced motor impairments and striatal oxidative stress. Notably, these effects were independent of mitochondrial complex II activity. In fact, probucol treatment, which did not change 3-NP-induced striatal mitochondrial complex II inhibition, protected against 3-NP-induced motor impairments and striatal oxidative stress, indicating that probucol was able to mitigate secondary events resulting from mitochondrial complex II dysfunction.

3-NP is a suicide inactivator of the mitochondrial complex II, directly resulting in mitochondrial dysfunction and ROS formation [41,42]. Thus, the 3-NP-based HD model is broadly accepted as a representation of the altered energy metabolism that occurs in this disorder [7]. In the present study, six 3-NP administrations induced a significant decrease in the body weights of the rats. This was consistent with previous studies [43,44]. Because probucol treatment did not change 3-NP-induced mitochondrial complex II inhibition but decreased 3-NP-induced body weight loss, it was possible to assume that 3-NP-induced body weight loss was not a direct consequence of complex II inhibition, but rather resulted from secondary events.

In our study, rats treated with 3-NP developed an impairment in the motor system, which was characterized by hypolocomotion in an open field test, and decreased the motor performance in the rotarod task. Alternatively, previous studies have described that the systemic administration of 3-NP (more than four doses) induces the onset of hypokinetic symptoms, while administration in two individual doses displayed hyperkinetic symptoms [45,46]. Thus, the 3-NP model can mimic and reproduce the hyperkinetic and hypokinetic symptoms of HD, depending on the time and dose administered, thereby enabling the examination of the initial (or early) and late phases of HD, respectively [7]. Thus, probucol treatment prevented 3-NP-induced motor impairments.

The clinical and behavioral symptoms of HD are commonly attributed to the loss of neurons, most prominently, the medium-spiny GABAergic neurons in the caudate nucleus and putamen, which show a progressive neuropathological change [47]. Similarly, 3-NP induces striatal toxicity, causing degeneration of GABAergic medium spiny neurons in the striatum, which resemble those processes observed in HD [48]. This neuronal death was correlated with motor dysfunction in 3-NP exposed animals [49,50]. The neurotoxin also appears to induce cell death via necrosis and apoptosis, which are processes also observed in HD. Interestingly, in the present study, 3-NP treatment did not induce caspase 3 activation, which indicated the absence of classical caspase-dependent apoptotic processes. Cirillo and colleagues also showed that subchronic treatment of 3-NP determined behavioral disabilities in the absence of striatal cell death [51]. Thus, 3-NP administration does not address the question of whether the behavioral changes are directly due to cell death or to the morpho-functional plasticity of the tripartite synapse following respiratory mitochondrial chain impairment [51].

In the present study, the behavioral alterations induced by 3-NP administration appear to be related to the significant reduction in complex II activity and an increase in oxidative stress in the striatum of rats, which may represent primary and secondary effects of the toxin. Importantly, our findings demonstrated that probucol administration did not change 3-NP-induced striatal mitochondrial complex II inhibition but attenuated both the behavioral alterations and striatal oxidative stress in 3-NP-treated rats, suggesting a potential link between these two events. Taken together, these results shed light on the molecular mechanisms mediating 3-NP-induced neurotoxicity, suggesting that 3-NP-induced oxidative stress is the main effect of complex II inhibition, which, in turn, results in behavioral deficits, as well as in oxidative damage in the striatum.

Knowledge of the role of oxidative stress in the pathogenesis of HD has grown within the past few years. Several studies have demonstrated the existence of oxidative damage in HD brains [4,52,53] and that antioxidants slow down disease progression [54,55]. Moreover, analyses of postmortem brain tissue from HD patients and brain tissues from animal models have shown increased levels of oxidative damage products [5,6]. In HD, the initial cause of oxidative insult is the presence of mutant Htt, which has been shown to increase levels of ROS in both neuronal and nonneuronal cells [56]. Elevated levels of MDA, a marker of lipid peroxidation, have also been shown in HD brains [57] and in R6/2 HD mouse brain [58,59]. Consistent with these studies, Chen and colleagues showed increased levels of MDA in the peripheral blood of HD patients [6]. Although increased lipid peroxidation products in HD blood has been previously demonstrated, whether the level of lipid peroxidation is correlated to disease severity is still unknown [60]. Accordingly, in the present study, we showed that i.p. 3-NP administration increased striatal lipid peroxidation. Our data were consistent with previous reports demonstrating that an increase in ROS on 3-NP exposure was accompanied by an increase in lipid peroxidation products [44,61,62].

In addition, the present study showed that catalase and SOD activities were increased in the striatum of animals exposed to 3-NP. These data suggested the existence of a compensatory mechanism that protected the cells from oxidative injury induced by the toxin. A similar event was also described in a transgenic mouse model of HD, in which the total SOD activity increased in young transgenic mice [63], as well as in human brain postmortem samples where an increase in SOD 2 and catalase activities was observed in the striatum [26]. In the same study, using western blotting analyses, the authors showed a significant increase in striatal SOD 2 levels [26]. SOD and catalase are both potent antioxidants in the cell’s defense mechanism. Superoxide anion (O_2_^•-^) is scavenged by SOD and converted to H_2_O_2_, which is transformed to water by catalase. Due to imbalances in mitochondrial function, increased generation of superoxide anion occurs [64]. Thus, it is reasonable to expect an upregulation of SOD and catalase activities in 3-NP-exposed rats in an attempt to decrease superoxide and H_2_O_2_ levels, thus preventing oxidative damage.

The role of oxidative stress in human HD or in HD animal models has been highlighted by the fact that most overexpressed proteins present antioxidant activity or mediate oxidative stress-related events [26,63]. Furthermore, the induction of SOD in response to oxidative stress has been well established in organisms, tissues, and cells growing under various stress conditions [65,66]. Based on such evidence, the protein levels of SOD 1 and 2 were evaluated using western blotting analyses in rat striatal tissues. However, the increase in SOD activity (Figure 5) did not correspond to changes in the protein levels (Figure S1A, B and C) in our experimental protocol. The potential post-transcriptional/allosteric regulation of striatal SOD activity in 3-NP treated animals represents an intriguing result that deserves further attention.

However, these events (upregulation of SOD and catalase activities Figure 5) were unable to prevent striatal lipid peroxidation (Figure 4) in 3-NP-treated rats. In this scenario, it is important to mention that GPx is an enzyme that, like catalase, detoxifies H_2_O_2_. Furthermore, probucol displayed a major effect in increasing striatal GPx activity (Figure 6C). In addition, some lines of evidence have shown that glutathione peroxidase, an enzyme important in mediating the detoxification of peroxides in several tissues [67], including the CNS [68], displays an important role in HD models and in the pathogenesis of HD. GPx and peroxide metabolisms appear to be important in HD because GPx was induced in the striatum and cortex of HD patients [26]. However, Chen and colleagues demonstrated decreased GPx activity in the erythrocytes of HD patients [6] and no change in GPx activity was observed in R6/1 mice, a transgenic model of HD [24]. However, this issue remains elusive and additional studies are well warranted. Although 3-NP treatment did not change GPx activity (this study), mice deficient in GPx were more vulnerable to the toxic effects of 3-NP [69]. These data reinforced the importance of GPx in oxidative stress conditions, suggesting that compounds that were able to enhance its activity may slow down oxidative damage and degenerative disease progression.

Although GPx has been reported as an enzyme important in the pathogenesis of HD [6,26], and hydrogen peroxide has been proposed as a critical ROS that mediates the deleterious effects observed in HD models [70], there have been no studies in the current literature on the potential protective effects of modulators of GPx activity in *in vivo* models of HD. To the best of our knowledge, this is the first *in vivo* study reporting the beneficial effects of this compound in an experimental model of HD that may be related, at least in part, to its positive modulating effects toward the GPx enzyme. This event was also investigated using western blotting analyses, which showed that probucol treatment did not change GPx-1 levels. This finding was reinforced by previous *in vitro* data demonstrating that probucol displayed a long-lasting protective effect against MeHg-induced neurotoxicity in cultured cerebellar granule cells [18]. Interestingly, this event was related to the positive modulator effects of probucol toward GPx-1 activity, suggesting that it modulates enzyme activity via direct activating effects with no changes in the enzyme levels [18]. Considering the significant probucol-induced increase in GPx activity, one might expected a decrease in GSH levels, as well as a consequential increase in GSSG (oxidized glutathione) levels. However, this effect was not observed in probucol-treated animals. Treatment with probucol induced an upregulation of GPx activity, which also displayed a major effect in increasing striatal GSH content. Our results were consistent with previous data showing that probucol was able to increase GSH levels in cardiac tissue [71]. GSH synthesis is regulated by the Nrf2 system, a transcription factor that regulates the basal and inducible expression of a wide array of antioxidant genes [72], including the first and key enzyme involved in GSH synthesis. Of particular importance, a recent study showed the upregulation of Nrf2 after systemic administration of probucol in rats [73]. Based on the aforementioned evidence and on our own data, it is reasonable to assume that the simultaneous increases in striatal GPx activity and GSH levels do not necessarily represent contradictory data because the mechanisms modulating both events are distinct: (i) probucol is a direct activator of GPx activity [18] and (ii) increased GSH levels in probucol-treated rats likely represent increased GSH synthesis via Nrf2 upregulation [73].

Based on this evidence, it is possible that the beneficial effects of probucol against 3-NP-induced oxidative damage and neurotoxicity are related, at least in part, to a modulating effect toward GPx. In this context, the induction of scavenging enzymes may attenuate the accumulation of ROS (particularly, H_2_O_2_), thereby protecting against potential cell injury and death. This idea is consistent with our data on striatal GPx activity (Figure 6C) and lipid peroxidation (Figure 4). However, it is likely that the direct antioxidant properties of probucol may also contribute to its beneficial effects against 3-NP-induced oxidative stress and motor impairment.

Probucol also reduced the increase in iNOS levels induced by 3-NP treatment. iNOS-like immunoreactivity and high levels of nitric oxide (NO) have been identified in the striatum of 3-NP-intoxicated animals [44,74]. iNOS is an enzyme that is commonly upregulated in response to inflammatory reactions and its persistent activation can lead to toxic levels of NO. At high concentrations, NO acts as a neurotoxin primarily due to its oxidative properties and ability to react with superoxide anions, thereby producing peroxynitrite (ONOO^-^), a reactive species that is highly toxic [75]. Furthermore, inflammation is a crucial factor involved in the toxicity elicited by 3-NP, and neuroinflammatory-like changes have been found in the striatum of rodents intoxicated with 3-NP [76]. Because probucol is an anti-inflammatory drug [13], the control of inflammation might represent an additional mechanism by which probucol affords neuroprotection in our experimental protocol.

In the present model, no alterations were observed in GFAP and caspase 3 expression. 3-NP has previously been shown to affect astrocytes by inducing increases in GFAP expression [51]. However, other studies have shown that chronic intoxication of rats with 3-NP induced a loss in the number of immunostained GFAP-positive cells in the striatum [77]. These data regarding the GFAP protein were observed in immunohistochemical studies in striatal sections, which revealed the presence of marked reactive astrocytosis, specifically in the caudate-putamen (CPu) and accumbens (Acb) striatal regions, as assessed by the intense GFAP staining [51]. Our negative data on GFAP expression in 3-NP-treated rats may be related to the methodology used (western blot), which did not discriminate between particular striatal structures.

In conclusion, the present findings indicated that probucol was able to counteract motor impairments and striatal oxidative stress induced by 3-NP administration in rats. Notably, these effects were independent of mitochondrial complex II activity because probucol treatment, which protected against motor impairments and striatal pro-oxidative damage, did not change 3-NP-induced striatal mitochondrial complex II inhibition, indicating that probucol was able to mitigate secondary events (i.e., increased ROS levels), which resulted from mitochondrial complex dysfunction. This evidence appears to be of great relevance when considering that mitochondrial dysfunction represents a ubiquitous event in several neurodegenerative diseases [78]. When extrapolating our data to humans, one may posit that people taking this drug may be less susceptible to the secondary events resulting from mitochondrial dysfunction. This renders probucol a promising molecule for further pharmacological studies searching for therapeutic strategies to slow down the progression of HD and other neurodegenerative disorders.

## Supporting Information

Figure S1 Effects of 3-NP and/or probucol on SOD 1, SOD 2 and GPx-1 expression. Treatments were conducted as previously mentioned (see Methods Section). SOD 1 (A), SOD 2 (B) and GPx-1 (D) levels were determined and expressed as optical density related to β-actin. The data are presented as the mean ± S.E.M. (n= 4 rats/group). (C) SOD 1 and SOD 2 representative western blot analysis. (D) GPx 1 representative western blot analysis.
(TIF)
