# Supplementary figures and images for: Probucol Increases Striatal Glutathione Peroxidase Activity and Protects against 3-Nitropropionic Acid-Induced Pro-Oxidative Damage in Rats
Source: PLoS One. 2013 Jun 14;8(6):e67658. doi: 10.1371/journal.pone.0067658 (PMC3683065; doi:10.1371/journal.pone.0067658)

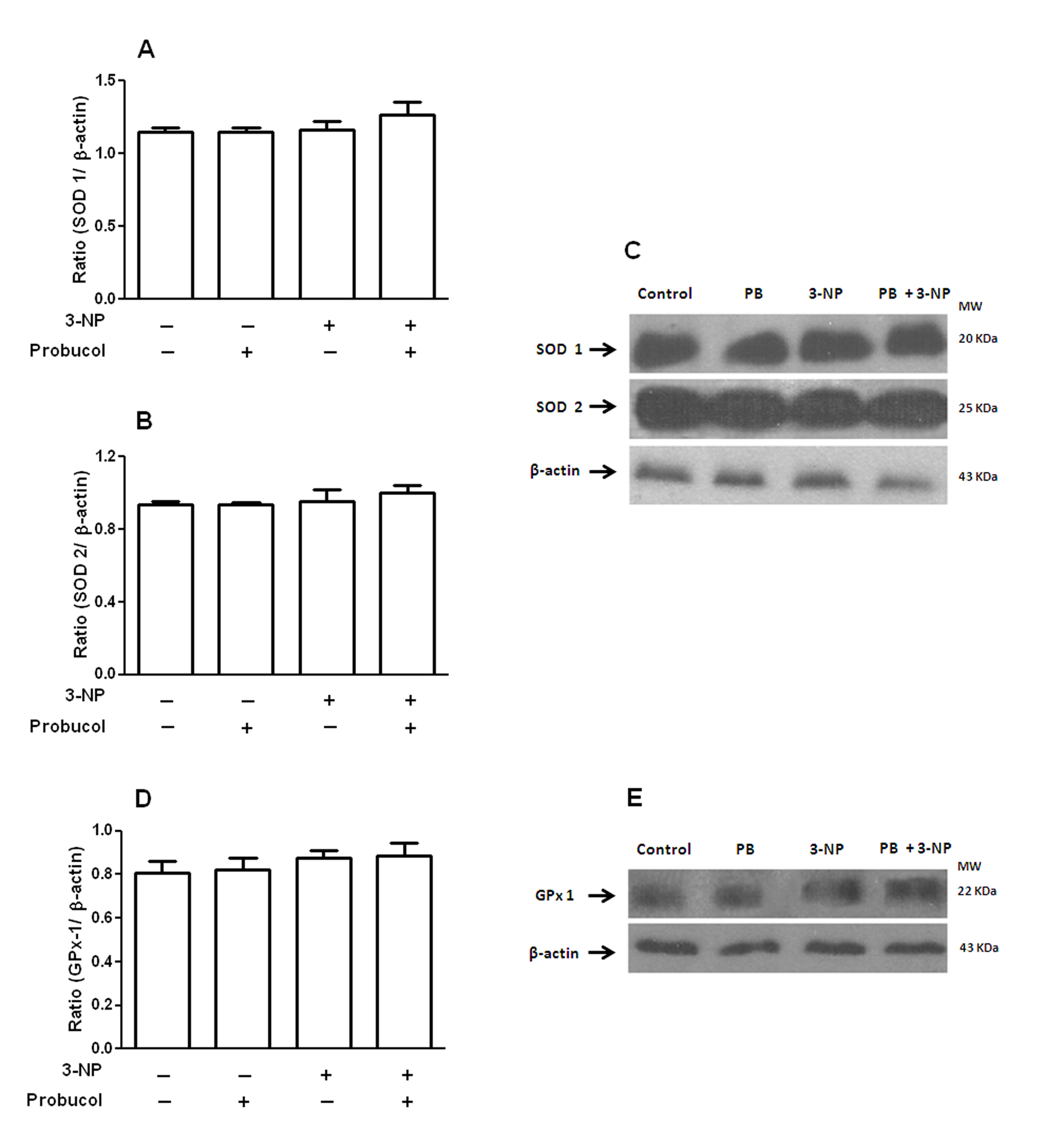

Supplement: Figure S1 — Treatments were conducted as previously mentioned (see Methods Section). SOD 1 (A), SOD 2 (B) and GPx-1 (D) levels were determined and expressed as optical density related to β-actin. The data are presented as the mean ± S.E.M. (n= 4 rats/group). (C) SOD 1 and SOD 2 representative western blot analysis. (D) GPx 1 representative western blot analysis. (TIF) [file pone.0067658.s001.tif]
